# Biosynthesized selenium nanoparticles to rescue coccidiosis-mediated oxidative stress, apoptosis and inflammation in the jejunum of mice

**DOI:** 10.3389/fimmu.2023.1139899

**Published:** 2023-02-17

**Authors:** Rewaida Abdel-Gaber, Maysar Abu Hawsah, Tahani Al-Otaibi, Ghada Alojayri, Esam M. Al-Shaebi, Osama B. Mohammed, Manal F. Elkhadragy, Saleh Al-Quraishy, Mohamed A. Dkhil

**Affiliations:** ^1^ Department of Zoology, College of Science, King Saud University, Riyadh, Saudi Arabia; ^2^ Department of Science and Technology, Al-Nairiyah University College, University of Hafr Al-Batin, Hafr Al-Batin, Saudi Arabia; ^3^ Department of Biology, College of Science, Princess Nourah bint Abdulrahman University, Riyadh, Saudi Arabia; ^4^ Department of Zoology and Entomology, Faculty of Science, Helwan University, Cairo, Egypt; ^5^ Applied Science Research Center, Applied Science Private University, Amman, Jordan

**Keywords:** coccidiosis, mice, nanoparticles, *Azadirachta indica*, oxidative stress, apoptosis, inflammation

## Abstract

One of the most crucial approaches for treating human diseases, particularly parasite infections, is nanomedicine. One of the most significant protozoan diseases that impact farm and domestic animals is coccidiosis. While, amprolium is one of the traditional anticoccidial medication, the advent of drug-resistant strains of *Eimeria* necessitates the development of novel treatments. The goal of the current investigation was to determine whether biosynthesized selenium nanoparticles (Bio-SeNPs) using *Azadirachta indica* leaves extract might treat mice with *Eimeria papillata* infection in the jejunal tissue. Five groups of seven mice each were used, as follows: *Group 1*: Non-infected-non-treated (negative control). *Group 2*: Non-infected treated group with Bio-SeNPs (0.5 mg/kg of body weight). *Groups 3-5* were orally inoculated with 1×10^3^ sporulated oocysts of *E. papillata*. *Group 3*: Infected-non-treated (positive control). *Group 4:* Infected and treated group with Bio-SeNPs (0.5 mg/kg). *Group 5*: Infected and treated group with the Amprolium. Groups 4 and 5 daily received oral administration (for 5 days) of Bio-SeNPs and anticoccidial medication, respectively, after infection. Bio-SeNPs caused a considerable reduction in oocyst output in mice feces (97.21%). This was also accompanied by a significant reduction in the number of developmental parasitic stages in the jejunal tissues. Glutathione reduced (GSH), glutathione peroxidase (GPx), and superoxide dismutase (SOD) levels were dramatically reduced by the *Eimeria* parasite, whereas, nitric oxide (NO) and malonaldehyde (MDA) levels were markedly elevated. The amount of goblet cells and *MUC2* gene expression were used as apoptotic indicators, and both were considerably downregulated by infection. However, infection markedly increased the expression of inflammatory cytokines (*IL-6* and *TNF-α*) and the apoptotic genes (*Caspase-3* and *BCL2*). Bio-SeNPs were administrated to mice to drastically lower body weight, oxidative stress, and inflammatory and apoptotic indicators in the jejunal tissue. Our research thus showed the involvement of Bio-SeNPs in protecting mice with *E. papillata* infections against jejunal damage.

## Introduction

The apicomplexan parasite genus *Eimeria* contains several species that cause coccidiosis in a range of animals (1). The sporulated oocyst, which is the infectious stage for *Eimeria* species, hatches to release eight sporozoites when a new host is ingested by these oocysts (2). Animal intestinal mucosa cells were invaded by sporozoites, which quickly split to create merozoites contained in a meront, resulting in tissue destruction and a severe local and systemic inflammatory reaction (3, 4). Decreased feed intake, reduced body-weight gain, dehydration, and higher vulnerability to other diseases are all consequences of *Eimeria* species infection (5–7).

Anticoccidial medications have long been used in conjunction with water and food to treat coccidiosis, but these methods are no longer thought to be viable due to rising parasite resistance, high prices, and concerns about residues in animal products (8, 9). However, because they are promising sources for new anti-parasitic candidate compounds, researchers were instructed to replace this parasite management program with plant-based natural remedies (10). These substances not only attack parasites but may also protect organs in target hosts that are parasite-infected (11). *Azadirachta indica*, also referred to as neem, nimtree, or Indian lilac, is a plant in the family Meliaceae. It is said to have health-promoting properties since it is a rich source of both non-isoprenoids and isoprenoids (especially azadirachtin) (12). Earlier investigators have confirmed their role as antibacterial (13), antifungal (14, 15), antimalarial (16), anti-inflammatory (17, 18), antiparasitic activities (19).

Currently, new trends such as nanotechnological approaches have been innovative antiparasitic agents (20). Since particles of nanoscale size exhibit unique properties that are distinct from both isolated atoms and bulk material, nanoparticles are attractive agents for the treatment of numerous parasite diseases (21, 22). For instance, selenium at the nanoscale has several advantageous benefits on health. According to Rayman (23), selenium nanoparticles (SeNPs) have antioxidant and anti-inflammatory properties. SeNPs are also known to have anti-leishmanial (24), and anti-coccidial (25, 26, 27) properties. Additionally, potential roles for SeNPs against murine schistosomiasis (28, 4), Giardiasis (29), and murine trichinosis (30), have been reported previously.

This study was designed to examine the ameliorative effect of biosynthesized SeNPs utilizing *Azadirachta indica* leaf extracts against intestinal *E. papillata* infection in mice based on the prior biological activities of neem.

## Materials and methods

### Experimental animals

Prior to the experiment, 35 male C57BL/6 mice were procured from the animal facility in the College of Pharmacy at King Saud University, when they were 10-12 weeks old and weighed 20-25 g. Parasitological examinations had been performed on all experimental mice to be free from parasites. All mice were given a regular feed as well as water *ad libitum*. Animals were kept in plastic cages under controlled conditions of temperature (23±5°C), humidity, and 12/12 hrs light-dark cycle. The week before the experiment, the animals were acclimated.

### Plant collection and extract preparation

Leaves of *Azadirachta indica* were gathered during summer 2022 from the botanical gardens in Riyadh, Saudi Arabia. The plant identification was verified by a taxonomist at the Department of Botany and Microbiology, College of Science (King Saud University) (KSU-10804). The *A. indica* leaves methanolic extract (AILE) was produced in accordance with Manikandan et al. (31).

### Plant extract analysis

The concentrations of phenolic and flavonoid contents were evaluated using the Folin–Ciocalteu technique and the aluminum chloride colorimetric method as described by Dkhil et al. (32) and determined as mg gallic acid/g dry leaves and mg Quercetin/g dry leaves, respectively.

### Synthesis of selenium nanoparticles

According to Vahdati and Moghadam (33), SeNPs were prepared by reducing sodium selenite (Nanocs Inc., Boston, MA, USA). Using a methanolic extract of *A. indica* in 9:1 ratio, an aqueous solution of Bio-SeNPs was prepared, warmed at 45 °C for 15 min, and then allowed to cool at 23±2 °C. Using a scanning electron microscope (SEM) (JSM-6060LV, JEOL) at an accelerating voltage of 15 kV, the size and morphology of the Bio-SeNPs were examined. Synthesis of Bio-SeNPs was confirmed by sampling the reaction mixture at regular intervals and the absorption maxima were scanned by UV-3600 Shimadzu spectrophotometer.

### Infection and treatment

This study used the coccidian parasite, *Eimeria papillata*, as a model parasite. *Eimeria* parasite oocysts were passaged in laboratory mice (*Mus musculus*). Unsporulated oocysts were collected from feces and allowed to sporulate in a solution containing 2.5% (w/v) potassium dichromate. They were then washed in phosphate buffer solution to prepare them for the remainder of the experiment.

Five groups of seven mice each were used, as follows: *Group 1*: Non-infected-non-treated (negative control). *Group 2*: Non-infected treated group with Bio-SeNPs (0.5 mg/kg of body weight). *Groups 3-5* were orally inoculated with 1×10^3^ sporulated oocysts of *E. papillata*, according to Abdel-Tawab et al. (34). *Group 3*: Infected-non-treated (positive control). *Group 4:* Infected and treated group with Bio-SeNPs (0.5 mg/kg) (26). *Group 5*: Infected and treated group with the Amprolium (120 mg/kg body weight) (35). Groups 4 and 5 daily received oral administration (for 5 days) of Bio-SeNPs and anticoccidial medication, respectively, after 60 min of infection.

The Bio-SeNPs dose was chosen based on our preliminary experiment for determining the best dose inhibiting oocyst shed in mouse faeces (see **Figure S1**), as well as our previous work (26).

Weight change was assessed on day 0 and day 5 of the experiment, according to Al-Quraishy et al. (36). Fecal pellets from each mouse in the 3^rd^, 4^th^, and 5^th^ groups were collected on the 5^th^ day post-infection (p.i.), and the total number of shed oocysts was determined in accordance with Schito et al. (37). All the mice were sacrificed, and the jejuna were harvested and kept for use in the experiment’s subsequent stages.

### Parasitic number and goblet cells

According to Adam and Caihak (38), portions of jejuna were fixed in 10% formalin for 24 hr, dehydrated and embedded in paraffin wax to be used for histological examinations. Hematoxylin-eosin (H&E) and Alcian blue staining of jejuna sections were employed to identify the parasite stages and goblet cells, respectively. Under an Olympus B×61 microscope (Tokyo, Japan), all slides were examined, photographed, and the number of parasitic stages and goblet cells were counted on at least ten well-orientated villous-crypt units (VCU).

### Immunohistochemistry

Jejunal sections were deparaffinized, treated with 3% H_2_O_2_ for 10 min, blocked with normal serum (5%), and then incubated overnight at 4 °C with a primary polyclonal rabbit anti-mouse antibody specific for cysteine aspartic acid protease-3 (Caspase-3) (1:100 dilution in PBS, Santa Cruz Biotechnology, CA, USA) (39). Samples were treated with a biotin-conjugated secondary antibody (1:2,000 dilution in PBS) after three PBS washes. The sections were counterstained with hematoxylin and re-incubated with streptavidin that was labeled with horseradish peroxidase and then examined and photographed using an Olympus B×61 microscope (Tokyo, Japan).

### Oxidative damage in mice jejunum

According to Tsakiris et al. (40), pieces of mice jejuna from all experimental groups were weighed and homogenized to produce 10% (*w/v*) homogenate in an ice-cold solution containing 300 mM sucrose and 50 mM Tris(hydroxymethyl)aminomethane-HCl (Sigma, St Louis, MO, USA). The homogenate was centrifuged at 500×g at 4 °C for 10 min. The supernatant was separated and used in a variety of biochemical analyses. According to the technique used by Beutler et al. (41), the concentrations of reduced glutathione (GSH) were calculated using the absorbance measurement at 405 nm and expressed as mg/g. The level of glutathione peroxidase (GPx) was assessed using the Pagila and Valentine (42) method, which reported values as U/g. Lipid peroxide by-product as malondialdehyde (MDA) contents (43) were estimated in the supernatant of jejunum homogenate at 534 nm and represented as nmol/g. The Montgomery and Dymock (44) procedure were used to measure the level of nitric oxide (NO), which was then represented as µmol/L. The quantity of superoxide dismutase (SOD) was quantified at 560 nm and displayed as U/g (45). All these parameters were measured using the proper kits (Biodiagnostic Co., Egypt), which were supported by the software of SoftMax^®^ Pro v. 6.3.1. The molecular device used for the analysis was the Spectra MAX 190.

### Gene expression analysis

Using Trizol (Invitrogen), total RNA was extracted from the preserved jejunal samples RNA later. Following the manufacturer’s instructions, RNA samples were digested with DNase (Applied Biosystems, Darmstadt, Germany) for at least 1 hr before being transformed into cDNA using the reverse transcription kit (Qiagen, Hilden, Germany). Using the suggested procedure, the QuantiTect^TM^ SYBR^®^ Green PCR kit (Qiagen, Hilden, Germany) was used to carry out the Real-time polymerase chain reaction **(**qRT-PCR). TaqMan 7500 system software v.1.2.3f2 (Applied Biosystems) was used to do relative quantitative evaluation of the amplification data. Using primers, the genes encoding the mRNAs for *mucin* (*MUC2*), *caspase-3* (*Casp3*), *B cell leukemia/lymphoma 2* (*BCL2*), interleukin-6 (IL-6), and tumor necrosis factor-alpha (TNF-α) were examined (see **Supplementary Table S1**). The PCR procedures followed those formerly outlined by Dkhil et al. (19). The Ct (2^−ΔΔCT^) method described by Livak and Schmittgen (46) was used to evaluate the differences between the mean of the gene expression and the reference gene was glyceraldehyde-3-phosphate dehydrogenase (GAPDH).

### Statistical analysis

We used one-way ANOVA to compare multiple variable comparisons, and the data is presented as the mean and standard deviation. We used Duncan’s test to compare the significance of the classes. The statistical significance level was set at p ≤ 0.05.

## Results

### Total phenolic and flavonoid contents

**Figure 1:**
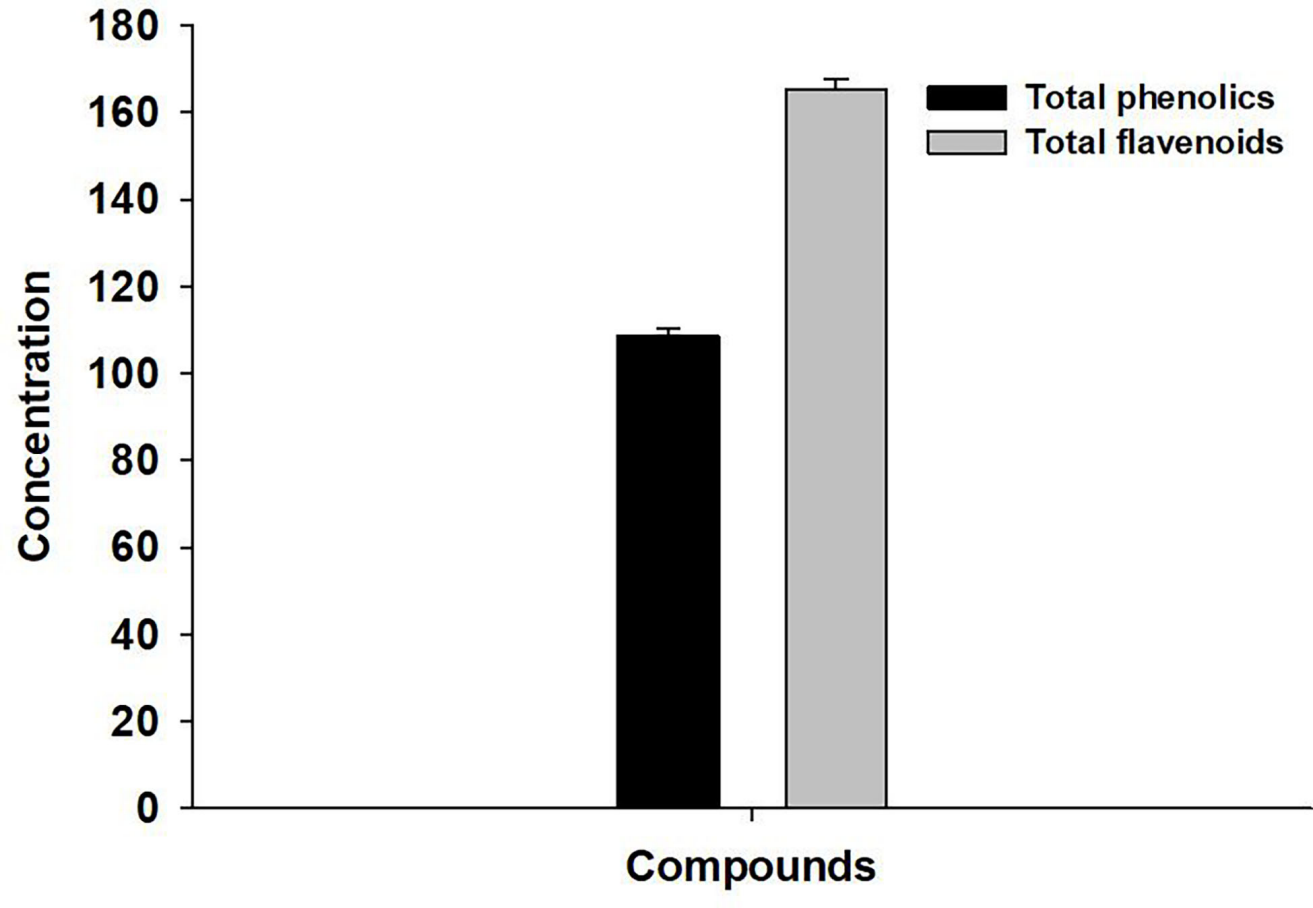
Concentration of phenolics (mg) and flavonoids (mg) in *A. indica* leaves extract.

The total concentration of phenolics and flavonoids in the investigated plant extract was found to be 108.76 ± 1.64 mg gallic acid/g dry leaves and 165.4 ± 2.25 mg quercetin/g dry leaves, respectively (**Figure 1**).

### Characterization of Bio-SeNPs

**Figure 2:**
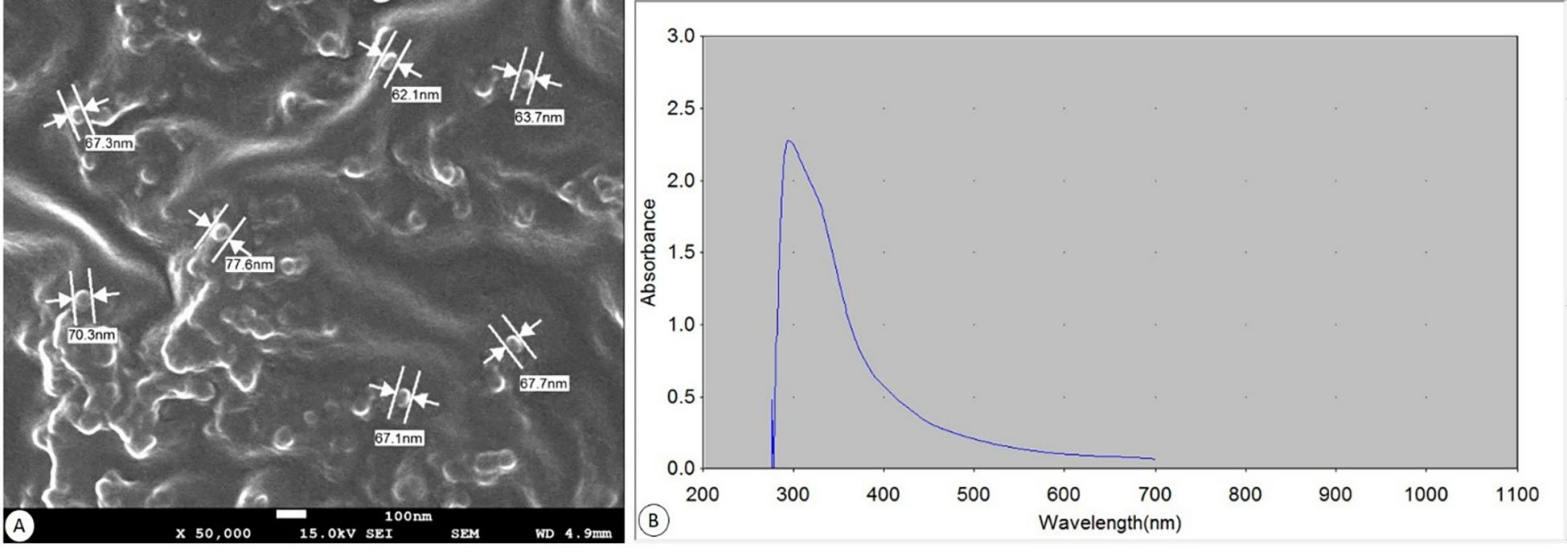
Biosynthesized SeNPs. **(A)** SEM at a magnification of ×50.000 (scale bar= 100 nm). **(B)** UV spectroscopic graph showing absorbance.

The SEM image (**Figure 2A**) revealed that the majority of the Bio-SeNPS has a spherical shape, where the size ranged from 62.1 to 77.6 nm with an average diameter of ∼67.97 nm. UV-visible absorption spectra of Bio-SeNPs showed that after 5 min of a reduction reaction had only one absorption peak at 308 nm (**Figure 2B**).

### Body weight change

**Figure 3:**
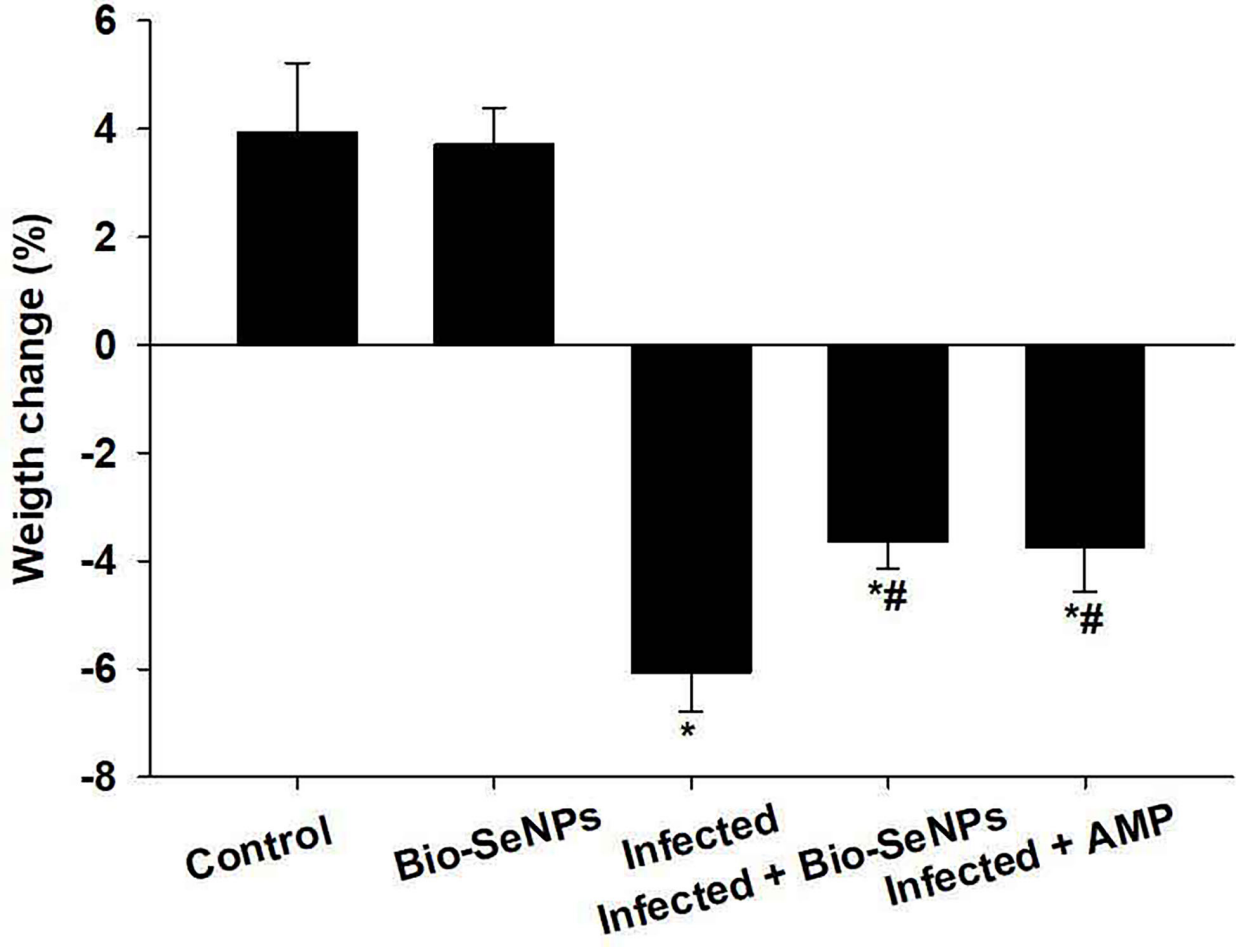
Bio-SeNPs improved weight loss due to infection with *Eimeria papillata* in mice. Values are means ± SEM. * significance (P ≤ 0.05) against non-infected control group, # significance (P ≤ 0.05) against infected group.

The non infected non treated control group grew normally resulting in an average weight gain of about 3.92% (**Figure 3**). Whereas, the experimentally infected group with *E. papillata* had lost their original weight by about 6.07%. After treatment with Bio-SeNPs, the loss of the original weight was reduced to -2.39% (**Figure 3**).

### Oocysts output

After 3 days following infection, all mice inoculated with 1×10^3^ sporulated *E. papillata* oocysts began excreting the oocysts with feces. The oocysts’ output varied between the infected-treated and infected-non treated groups on day 5 post-infection (p.i.). In the infected group, there were around 5.130 × 10^6^ ± 0.38 × 10^6^ oocysts shedding per g of feces. On day 5 p.i., the Bio-SeNPs treatment significantly reduced the shedding rate, which was 0.148 × 10^6^ ± 0.87 × 10^5^ oocysts/g feces (∼ 97.21%) compared to 0.138 × 10^6^ ± 0.52 × 10^5^ oocysts/g feces with the reference anticoccidial drug.

### Histological and immunohistochemical studies

**Figure 4:**
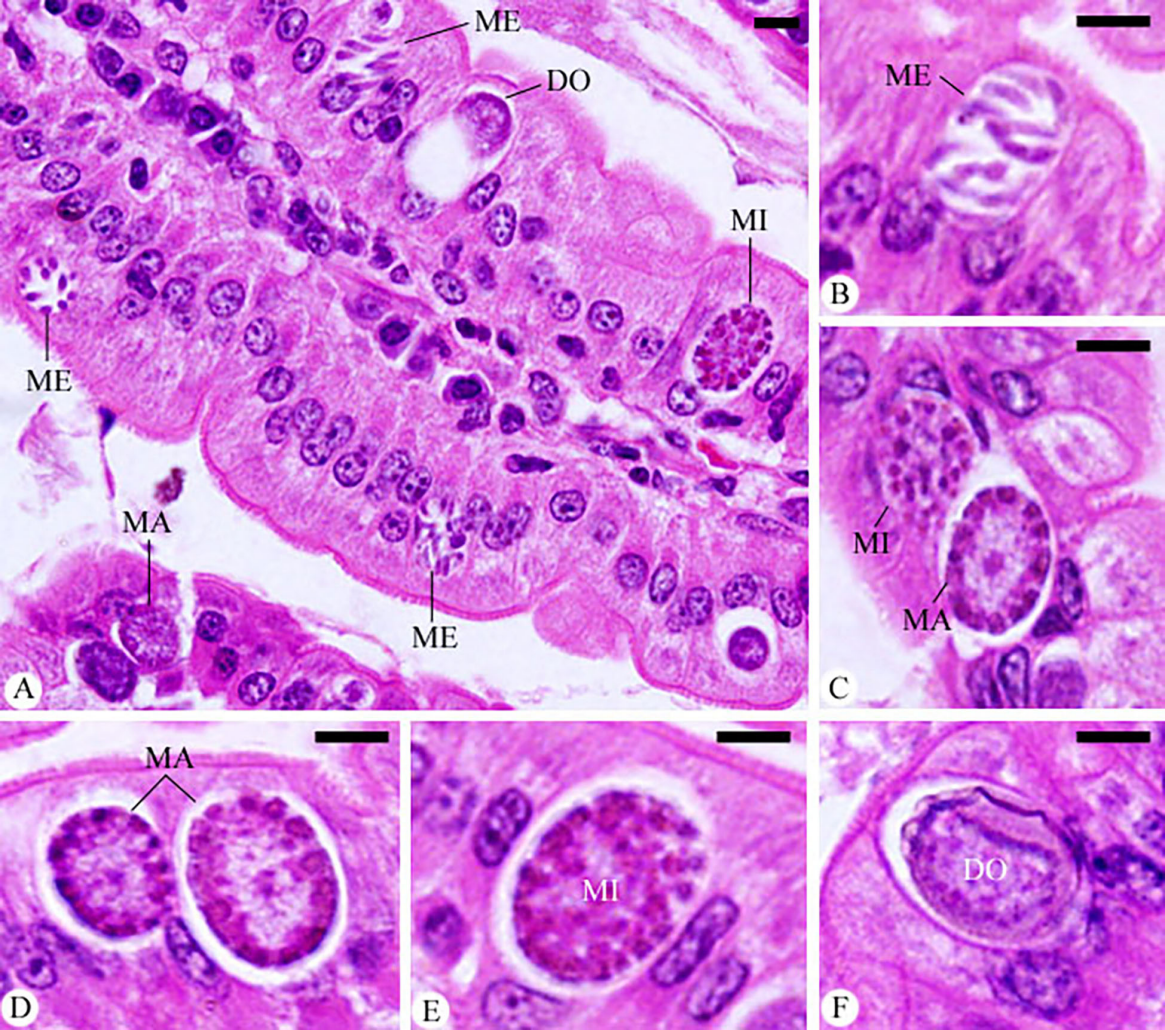
Infected jejunum with *Eimeria papillata* on day 5 p.i. **(A)** different developmental stages. **(B)** meronts (ME). **(C)** macrogamont (MA) and microgamont (MI). **(D)** macrogamont (MA). **(E)** microgamont (MI). **(F)** developing oocyst (DO). Sections were stained with hematoxylin and eosin (H&E). Scale bar= 25µm.

**Figure 5:**
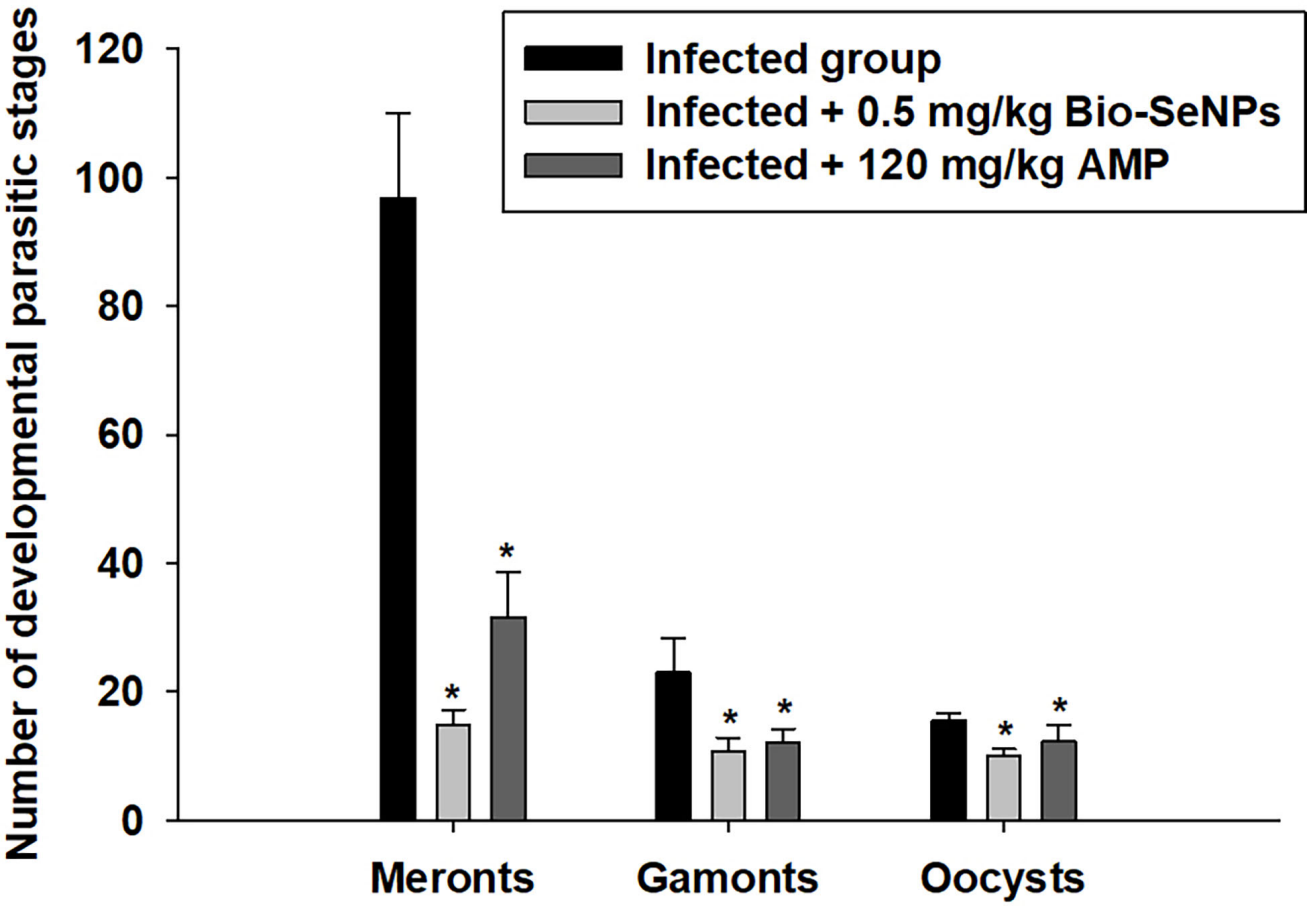
The number of developmental parasitic stages of *Eimeria papillata* in jejunum among the infected and treated groups. All values are means ± SD. ^*^ significance (P ≤ 0.05) between the infected and treated groups.

Meronts, microgamonts, macrogamonts, and developing oocysts were developed in the epithelial cells of the jejunum in experimentally infected of mice with *E. papillata* oocysts, as observed in the hematoxylin-eosin-stained sections (**Figure 4**). In the jejunal villi of the infected mice, these stages were counted per 10 VCUs as 96.83 ± 13.19 meronts, 23.01 ± 5.40 gamonts, and 15.52 ± 1.08 developing oocysts (**Figure 5**). Remarkably, the number of meronts and male and female gamonts were significantly reduced following treatment with Bio-SeNPs by 14.86 ± 2.38 and 10.83 ± 2.04, respectively, compared to the reference drug’s values of 31.63 ± 7.02 and 12.22 ± 2.54 (**Figure 5**).

**Figure 6:**
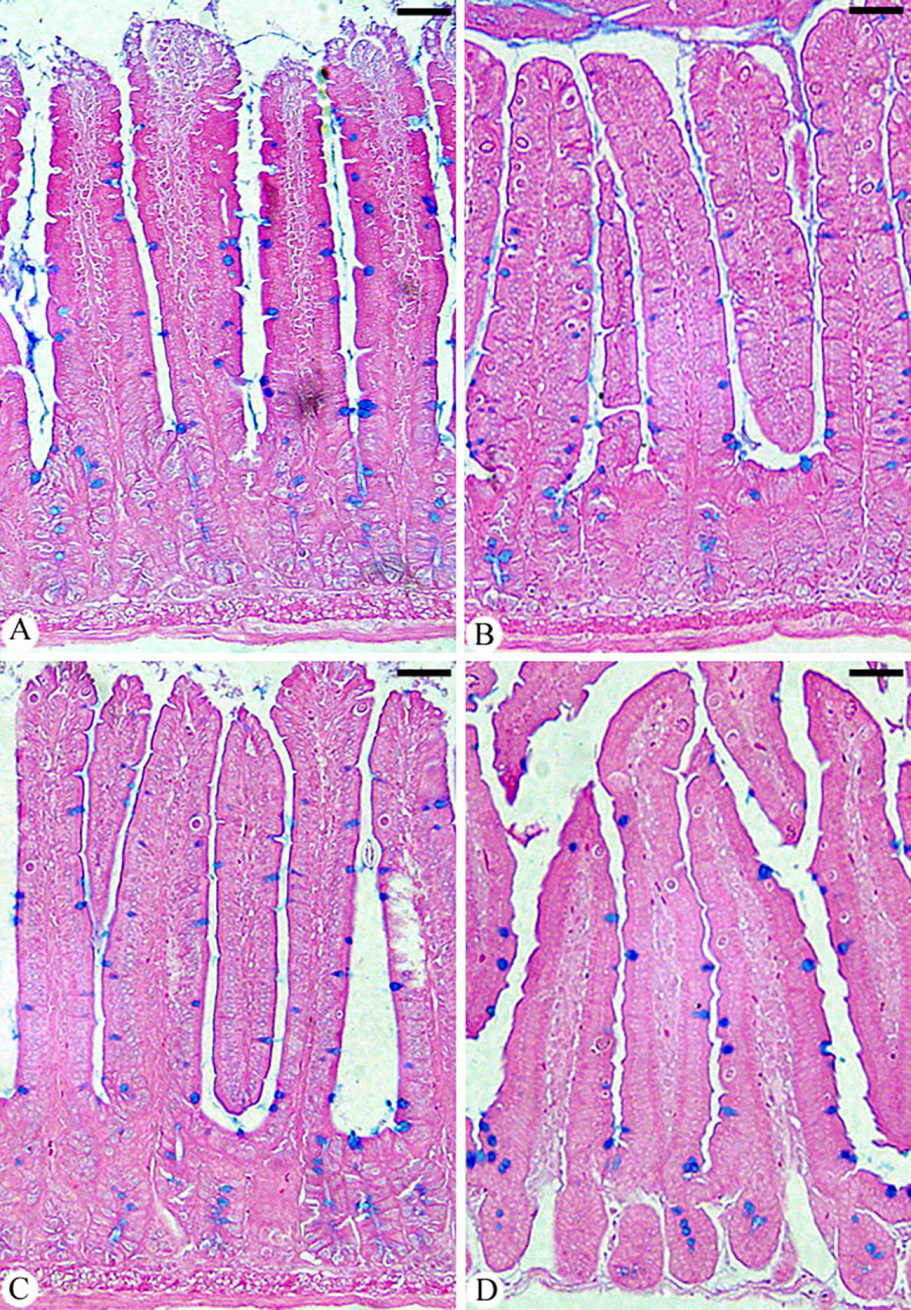
Jejunum sections showing goblet cells in **(A)** control group, **(B)** infected group, **(C)** infected group treated with Bio-SeNPs, and **(D)** infected group treated with AMP. Sections were stained with alcian blue and counted with eosin goblet cells in 10 well-oriented villus-crypt units (VCU). Scale bar = 100µm.

**Figure 7:**
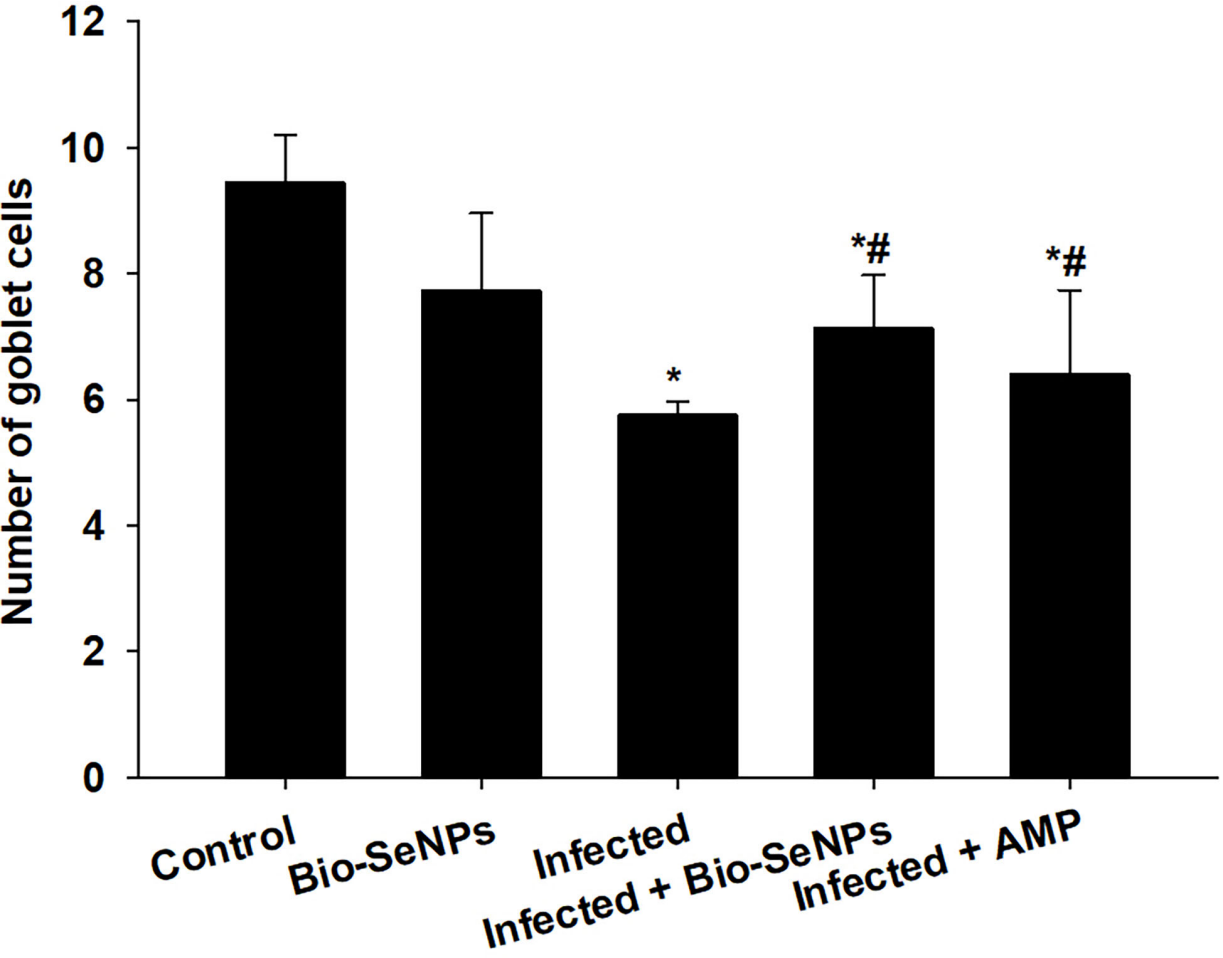
Changes in the number of jejunal goblet cells in the control group, infected group, treated group with Bio-SeNPs and the infected group treated with Bio-SeNPs and AMP. Values are means ± SEM. ^*^ significant change concerning the control group, ^#^ significant change concerning the infected group.

Microscopic analysis of jejunal sections stained with alcian blue (**Figure 6**) showed that mice infected with *E. papillata* had significantly fewer goblet cells (5.75 ± 0.22/VCU) than the non-infected group (9.44 ± 0.75/VCU) (**Figure 7**). The number of goblet cells was significantly higher in mice treated with Bio-SeNPs (7.13 ± 0.83/VCU) than in mice treated with a reference drug (6.40 ± 1.32/VCU) (**Figure 7**).

**Figure 8:**
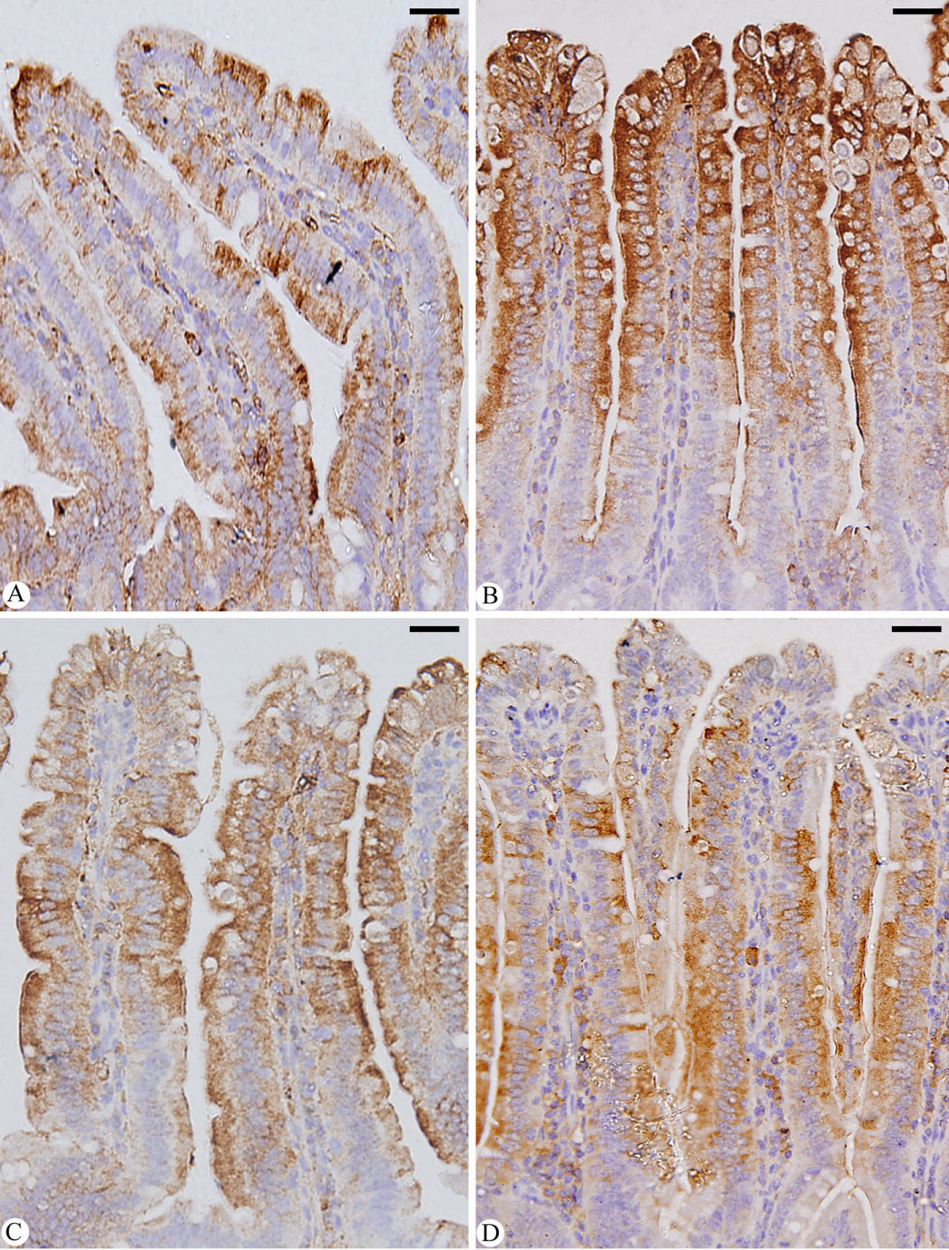
Immunohistochemical localization of CASP-3 in the jejuna of mice. **(A)** control non-infected jejunum. **(B)** *E. papillata* infected jejunum with an increased number of CASP-3 positive cells. **(C, D)** infected treated mouse (Bio-SeNPs and AMP, respectively) with decreased number of CASP-3 positive cells. Scale bar = 100µm.

When mice were infected with *E. papillata*, jejunal sections from the control, infected and treated groups were stained for caspase-3 expression. This showed that the infection caused a qualitative elevation of the caspase-3 expression level compared to the non-infected group (**Figure 8**). After Bio-SeNPs treatment, the expression of caspase-3 significantly changed in contrast to the infected groups (**Figure 8**).

### Effect of Bio-SeNPs on oxidative stress in jejunal mice

In the infected group, the level of GSH significantly decreased from 61.37 ± 2.93 in the control group to 34.16 ± 3.49 mg/g. In contrast, mice treated with Bio-SeNPs and reference drug had increased GSH levels, reaching 59.84 ± 4.76 and 58.70 ± 5.84 mg/g, respectively (**Table 1**). Additionally, the level of GPx significantly decreased in the infected group from 29.82 ± 6.08 in the control group to 13.29 ± 2.49 U/g. As opposed to this, mice treated with Bio-SeNPs and reference drug had increased in their GPx levels of 21.07 ± 1.37 and 20.42 ± 1.28 U/g, respectively (**Table 1**).

**Table 1:** Effect of Bio-SeNPs on the value of glutathione reduced (GSH), malondialdehyde (MDA), nitric oxide (NO), superoxide dismutase, and glutathione peroxidase (GPx) of mice infected with *E. papillata*.

Group	GSH (mg/g tissue)	MDA (nmol/g tissue)	NO (µmol/g tissue)	SOD (U/g tissue)	GPx (U/g tissue)
**Control**	61.37 ± 2.93	18.91 ± 1.5	8.89 ± 0.82	4.50 ± 0.32	29.82 ± 6.08
**Bio-SeNPs**	59.56 ± 3.37	19.62 ± 1.05	8.63 ± 3.14	4.89 ± 0.67	31.35 ± 3.72
**Infected**	34.16 ± 3.49 ^*^	42.32 ± 4.16 ^*^	20.77 ± 3.05 ^*^	3.05 ± 0.31 *	13.29 ± 2.49 ^*^
**Infected + Bio-SeNPs**	59.84 ± 4.76 ^#^	22.17 ± 4.44 ^#^	10.26 ± 2.89 ^#^	4.17 ± 0.49 #	21.07 ± 1.37 ^*,#^
**Infected +AMP**	58.70 ± 5.84 ^#^	24.53 ± 5.83 ^#^	10.94 ± 0.45 ^*,#^	4.29 ± 0.46 ^#^	20.42 ± 1.28 ^*,#^

^*^ indicates a significance against the control group with p< 0.001 and ^#^ indicates a significance against the infected group with p< 0.01.

Compared to 8.89 ± 0.82 µmol/g in the control group, the *E. papillata* infection caused a highly significant increase in jejunal NO to 20.77 ± 3.05. However, NO levels were significantly reduced after treatment with a dosage of Bio-SeNPs and reference drug, reduced to 10.26 ± 2.89 and 10.94 ± 0.45 µmol/g, respectively (Table 1). The MDA level was significantly increased in the infected group, going from 18.91 ± 1.59 in the control group to 42.32 ± 4.16 nmol/g. In contrast, the MDA levels of mice treated with Bio-SeNPs and reference drug were significantly reduced to 22.17 ± 4.44 and 24.53 ± 5.83 nmol/g, respectively (**Table 1**).

In the infected group, the SOD level significantly decreased from 4.50 ± 0.32 in the control group to 3.05 ± 0.31 U/g. In contrast, mice treated with Bio-SeNPs and reference drug had significantly lower levels of SOD, measuring 4.17 ± 0.49 and 4.29 ± 0.46 U/g, respectively (**Table 1**).

### Effect of Bio-SeNPs on mRNA expression

**Figure 9:**
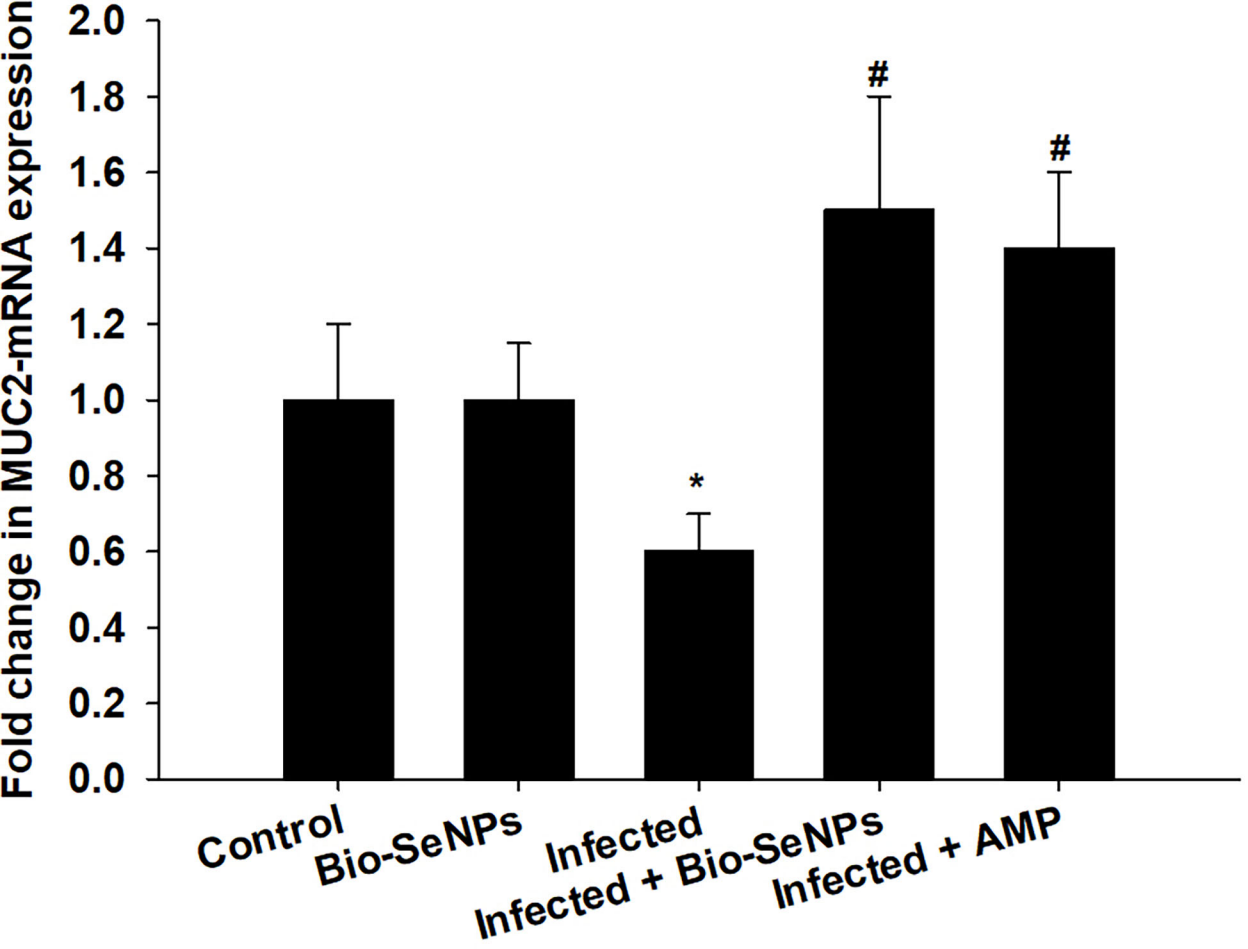
Effect of Bio-SeNPs on the mRNA expression of MUC2 in the jejunal samples from *E. papillata*-infected mice. The expression values obtained by RT-PCR analysis were normalized to the reference gene GAPDH mRNA level and are shown as fold induction (in log 2 scale) relative to the mRNA level in the control. * significance changes concerning the control group, # significance changes concerning the infected group.

The *MUC2* gene’s expression level was downregulated because of *Eimeria papillata* infection, according to qRT-PCR (**Figure 9**). However, using Bio-SeNPs significantly upregulated the expression of the *MUC2* gene from 0.60 to 1.50 fold (**Figure 9**).

**Figure 10:**
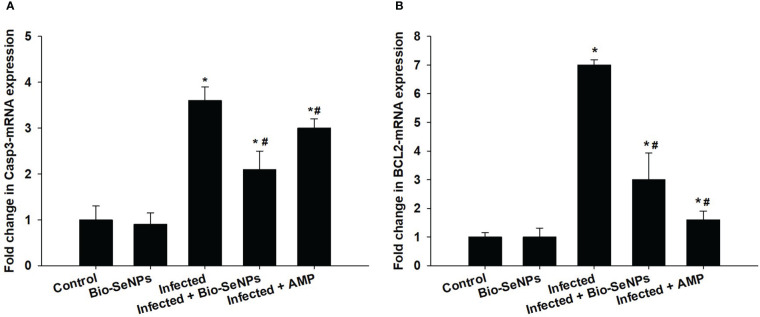
Effect of Bio-SeNPs on the mRNA expression of *Caspase-3* **(A)** and *BCL2* **(B)** in the jejunal samples from *E. papillata*-infected mice. The expression values obtained by RT-PCR analysis were normalized to the reference gene GAPDH mRNA level and are shown as fold induction (in log 2 scale) relative to the mRNA level in the control. ^*^ significance changes concerning the control group, ^#^ significance changes concerning the infected group.

*BCL2* and *Caspase-3* gene expression were upregulated because of the infection induced by *E. papillata* (**Figure 10**). After Bio-SeNPs treatment, *caspase-3* gene expression was downregulated significantly from 3.60 to 2.10 fold, while, *BCL2* gene expression was downregulated from 7.0 to 3.0 fold (**Figure 10**).

**Figure 11:**
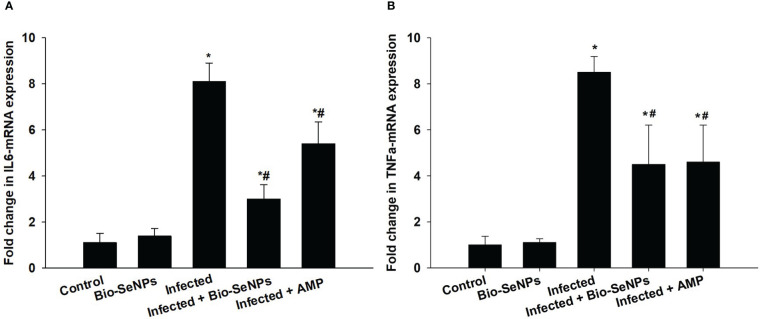
Effect of Bio-SeNPs on the mRNA expression of IL-6 **(A)** and TNF-α **(B)** in the jejunal samples from *E. papillata*-infected mice. The expression values obtained by RT-PCR analysis were normalized to the reference gene GAPDH mRNA level and are shown as fold induction (in log 2 scale) relative to the mRNA level in the control. ^*^ significance changes concerning the control group, ^#^ significance changes concerning the infected group.

Additionally, the expression of the IL-6 and TNF-α was upregulation because of *E. papillata* infection (**Figure 11**). When compared to the non-infected control group, this increase in mRNA expression of these genes was about 8.10 and 8.50, respectively (**Figure 11**). Treatment with Bio-SeNPs resulted in a 3.0 and 4.50 fold, respectively, with a significant downregulation of IL-6 and TNF-α (**Figure 11**).

## Discussion

More than 30 sulfanilamide drugs have been used for the prevention and treatment of coccidiosis since the 1940s. These anticoccidial agents have an effect on *Eimeria* species’ cofactor synthesis, mitochondrial functions, or cell membrane function (47). The need for innovative anti-*Eimeria* medications is justified by growing worries about parasite resistance, consumer health, and the environmental safety of commercial drugs (11). Nanopartic les now open new opportunities for novel medical therapies to combat parasite infection (26, 48). For instance, nano-selenium (SeNPs) was used against leishmaniasis (24), schistosomiasis (28), giardiasis (29), and murine trichinosis (30). For instance, selenium at the nanoscale has several advantageous benefits on health. According to Rayman (23), SeNPs have antioxidant and anti-inflammatory properties. SeNPs are also known to have anti-leishmanial (24), and anti-coccidial (25, 26, 27) effects. They have demonstrated antimicrobial, antiviral, and anti-parasitic properties (27, 49, 50). This study investigates the potential anti-coccidiosis effects of Bio-SeNPs utilizing *Azadirachta indica* leaves extract, which is likely due to the extract’s composition and SeNPs’ unique properties.

In addition to having a decreasing effect on the intracellular stages of *E. papillata* in the jejunum, the Bio-SeNPs were an efficient agent in reducing the total number of shed oocysts on the 5^th^-day after infection in mice by around 97.21%. Most anti-coccidial therapies are known to cause this suppression of intracellular *Eimeria* stages and reducing influence on oocyst shedding rate. High levels of polyphenolic compounds found in the plant extract (51, 52) may be responsible for the anticoccidial properties. These compounds have a potent antimicrobial effect by interfering with microbial cell walls and membranes, altering their permeability for cations and water, which causes impaired membrane functions and leakage of cellular constituents and finally to cell death (53–56).

Our finding showed that *E. papillata* infection led to weight loss. Previous studies attributed this loss brought on by the consumption of the parasitic *Eimeria* stages to the intestinal epithelial carbohydrate content and the severe structural alterations that resulted (57–59). Due to the presence of bioactive compounds in the plant extract, which helped to enhance the jejunal histological architecture and restore the mice’s nutritional condition, a considerable improvement in body weight was observed after treatment.

A strong inflammatory response and oxidative damage to the mice jejunum are linked to *E. papillata* infection. Bio-SeNPs treatment of the infected animals demonstrated an excellent modulation of the oxidative damage of mice jejunum tissue. The strong potential effect of Bio-SeNPs is due to the antioxidant (25, 27, 60–63) and anti-inflammatory (19, 26, 64–67) activities of both SeNPs and components of the plant extract.

An imbalance between endogenous antioxidant defense and free radical production is brought on by the coccidial infection (68, 69). Our findings showed that *E. papillata* infection is associated with oxidative damage to the mice jejunum, which causes the antioxidant enzymes to be depleted and GSH, GPx, and SOD levels to be reduced. These oxidative parameters are crucial for shielding the animal body from free radical damage during *Eimeria* infection. According to Georgieva et al. (68)Dkhil et al. (, 70), and Elmahallawy et al. (71), antioxidant enzymes use nanoparticles active sites to detoxify the accumulation of the reactive oxygen species that causes destructive changes in the jejunum epithelia. Bio-SeNPs significantly prevent the infection-induced loss of these markers and upregulated their activity, which is typically decreased during oxidative damage induced by infection.

The synthesis of several carbonyl compounds, such as MDA, results from the oxidation of lipid peroxides (11, 25, 72–74). MDA levels decrease following treatment with SeNPs or neem leaf extract. Al-Quraishy et al. (36) reported that NO level upregulated as an immunological response to the presence of the infective sporozoite stages that invade the jejunal epithelial cells, causing inflammation. Furthermore, the unbalanced status led to the elevation of nitric oxide levels because of infection, which is consistent with Dominquez et al. (75). By increasing NO levels, the Bio-SeNPs could dramatically reduce the severity of the infection in the jejunum. This illustrates the potential antioxidant function of Bio-SeNPs. According to prior investigations, active compounds such as flavonoids and other derivatives were present in SeNPs and plant extracts (14, 76–78). In this study, the biological function of flavonoids is indicated by their presence in Bio-SeNPs.

One of the major intestinal immune-competent cells, goblet cells secrete mucus, which is regarded as a protective barrier (79). Multipotential stem cells can be detected in the jejunal villi. The reduced number of goblet cells could be a result of the parasite causing damage to the multipotent stem cell population at the base of the Lieberkühn crypts (80). This study demonstrated how the *Eimeria*-induced infection decreased the number of goblet cells in the mice jejunum. According to Yunus et al. (81), the alteration in goblet cells may affect how susceptible the *Eimeria*-infected host is to be limiting the parasite’s ability to grow or penetrate the local epithelium. Through the restoring functions of stem cells, Bio-SeNPs were able to increase the number of jejunal goblet cells infected with *E. papillata*. Additionally, goblet cells are primarily in charge of producing and maintaining the mucus barrier through the synthesis of mucin and are greatly influenced by interactions with the immune system (82, 83). This study demonstrated how *E. papillata* infection decreased the MUC2 gene’s expression level. According to this study by Boltin et al. (84), the MUC2 is one of the primary secreted, gel-forming mucin components that helps the mucus barrier form. Treatment with Bio-SeNPs significantly increased the expression of the MUC2 gene, supporting the findings of Gum et al. (85) and Larsson et al. (86), which suggested that an improvement in goblet cell activity may influence the regulation of mucin production.

A considerable elevation in *IL-6* and *TNF-α* indicated the mice’s evident systemic inflammatory response to the *E. papillata*-induced infection. Pro-inflammatory cytokines including *IL-6 and TNF-α* have been found to control mucin production, according to Enss et al. (87). In contrast to the current study, cytokine inflow and mucin glycosylation downregulation were brought on by the *Eimeria* parasite infection. According to Hsu et al. (88), this negative association is frequently linked to a decrease in goblet cells, a thickening of the mucus, and the rate at which mucin is transported from the Golgi to secretory vesicles. Bio-SeNPs therapy dramatically reduced the systemic inflammatory response in infected mice. Our findings demonstrated that Bio-SeNPs protect host tissues by acting as anti-inflammatory agents in addition to targeting Eimeria stages within infected tissues. According to Oke et al. (52) and Abdel-Moneim et al. (63), polyphenolic chemicals may be responsible for this anti-inflammatory effect. Previous research demonstrated the immunomodulatory and anti-inflammatory properties of flavonoids as well as their capacity to reduce the production of pro-inflammatory cytokines (89, 90). Apoptosis may control the host’s response to certain intracellular parasitic diseases and aid in the removal of damaged or infected cells (91, 92). The pro-apoptotic genes BCL2 and caspase-3 significantly upregulated in this study, indicating the death of mouse jejunal cells. Previous investigations found an increase in the quantity of apoptotic cells in the jejuna of mice infected with *E. papillata* (6, 93). The eimeriosis-induced apoptotic alterations in jejunal cells might be improved by the Bio-SeNPs. Previous studies by Alkhudhayri et al. (27)Schumacher et al. (, 94), and Mehanna et al. (95) all reported SeNPs’ anti-apoptotic action.

## Conclusion

The combined effects of Bio-SeNPs against *E. papillata*-induced infection in C57BL/6 male mice result in considerable anticoccidial activity, a significant improvement in the histological changes of the mice jejunum, antioxidant status, anti-apoptotic, and anti-inflammatory activities. To understand the mechanism of the Bio-SeNPs’ action on both the parasite and the host mice, more research is necessary.

## Data availability statement

The original contributions presented in the study are included in the article/**Supplementary Materials**, further inquiries can be directed to the corresponding author/s.

## Ethics statement

This research was approved by the Research Ethics Committee (REC) at King Saud University (approval number KSU-SE-22-66).

## Author contributions

RA, SA and MAD designed the study. RA, MA, TA, GA, EMA, OBM, MFE, SA and MAD carried out the experiments and analyzed the data. All authors contributed to the article and approved the submitted version.

## Supplementary material

Supplementary Figure 1 Oocyst output in mice infected with *Eimeria papillata* and for infected treated groups with 0.25 mg/kg, 0.5 mg/kg, 1 mg/kg Bio-SeNPs, and 120 mg/kg amprolium (AMP) on day 5 p.i. All values are means ± SD. * significance (P ≤ 0.05) between infected and treated groups.
