# Supplementary figures and images for: Biosynthesized selenium nanoparticles to rescue coccidiosis-mediated oxidative stress, apoptosis and inflammation in the jejunum of mice
Source: Front Immunol. 2023 Feb 17;14:1139899. doi: 10.3389/fimmu.2023.1139899 (PMC9982015; doi:10.3389/fimmu.2023.1139899)

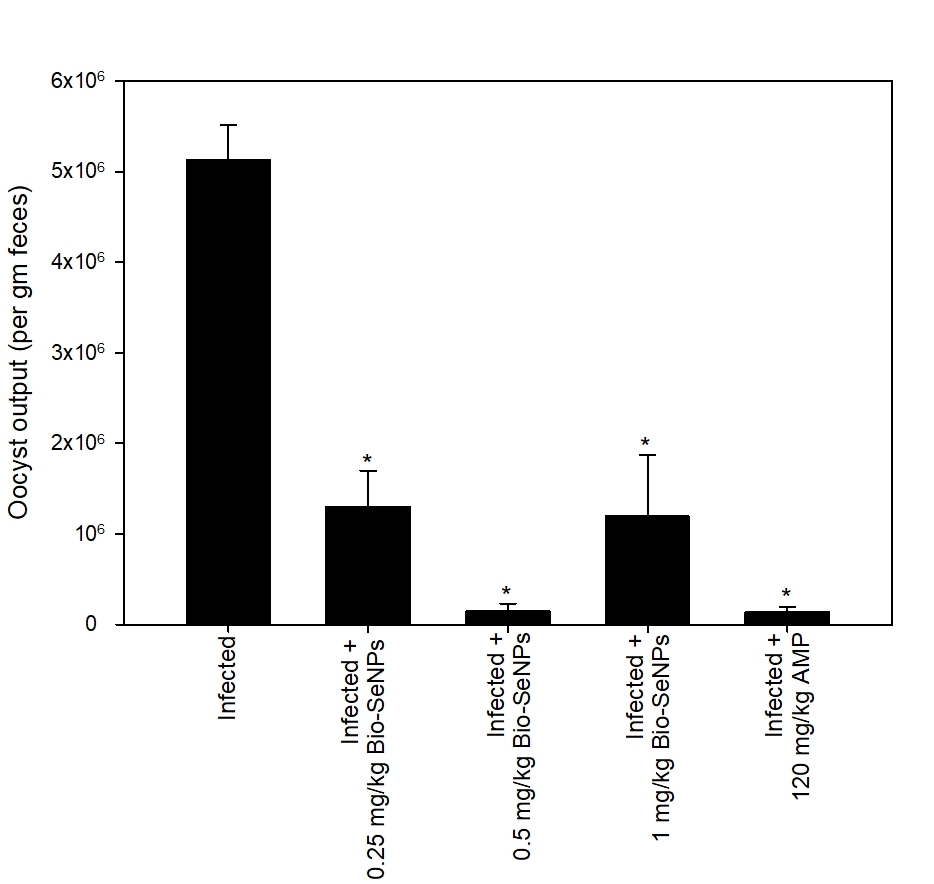

Supplement: Supplementary Figure 1 — Oocyst output in mice infected with Eimeria papillata and for infected treated groups with 0.25 mg/kg, 0.5 mg/kg, 1 mg/kg Bio-SeNPs, and 120 mg/kg amprolium (AMP) on day 5 p.i. All values are means ± SD. * significance (P ≤ 0.05) between infected and treated groups. [file Image_1.jpeg]
