# Supplementary material for: Biosynthesized selenium nanoparticles to rescue coccidiosis-mediated oxidative stress, apoptosis and inflammation in the jejunum of mice
Source: Front Immunol. 2023 Feb 17;14:1139899. doi: 10.3389/fimmu.2023.1139899 (PMC9982015; doi:10.3389/fimmu.2023.1139899)
Supplement: Supplementary file 2 [file Table_1.docx]

**Table S1**: Primers used for the gene encoding the mRNAs for some selected proteins

| **Gene** | **Type** | **Primer sequence (5' →3')** |
| --- | --- | --- |
| Mucin (muc2) | Forward | TTCGGCACGAGCAACTTTG |
|  | Reverse | GGCAGGACACCTTGTCATTG |
| Caspase 3 (Casp3) | Forward | GGGGAGCTTGGAACGCTAAG |
|  | Reverse | CCACTGACTTGCTCCCATGT |
| B cell leukemia/lymphoma 2 (Bcl2) | Forward | AGCATGCGACCTCTGTTTGA |
|  | Reverse | GCCACACGTTTCTTGGCAAT |
| Interleukin 6 (IL6) | Forward | CTGCAAGAGACTTCCATCCAG |
|  | Reverse | AGTGGTATAGACAGGTCTGTTGG |
| Tumor necrosis factor-alpha (TNF-𝛼) | Forward | ACCCTCACACTCACAAACCA |
|  | Reverse | ACCCTGAGCCATAATCCCCT |
| Glyceraldehyde-3-phosphate dehydrogenase (GAPDH) | Forward | CCCTTAAGAGGGATGCTGCC |
|  | Reverse | ACTGTGCCGTTGAATTTGCC |
